# Evolutionary dynamics of the Trivers–Willard effect: A nonparametric approach

**DOI:** 10.1002/ece3.8012

**Published:** 2021-08-17

**Authors:** Matthias Borgstede

**Affiliations:** ^1^ University of Bamberg Bamberg Germany

**Keywords:** continuous strategies, evolutionary dynamics, evolutionary invasion analysis, integral projection models, Trivers–Willard hypothesis, two‐sex models

## Abstract

The Trivers–Willard hypothesis (TWH) states that parents in good condition tend to bias their offspring sex ratio toward the sex with a higher variation in reproductive value, whereas parents in bad condition favor the opposite sex. Although the TWH has been generalized to predict various Trivers–Willard effects (TWE) depending on the life cycle of a species, existing work does not sufficiently acknowledge that sex‐specific reproductive values depend on the relative abundances of males and females in the population. If parents adjust their offspring sex ratio according to the TWE, offspring reproductive values will also change. This should affect the long‐term evolutionary dynamics and might lead to considerable deviations from the original predictions.

In this paper, I model the full evolutionary dynamics of the TWE, using a published two‐sex integral projection model for the Columbian ground squirrel (*Urocitellus columbianus*). Offspring sex ratio is treated as a nonparametric continuous function of maternal condition. Evolutionary change is treated as the successive invasion of mutant strategies. The simulation is performed with varying starting conditions until an evolutionarily stable strategy (ESS) is reached.

The results show that the magnitude of the evolving TWE can be far greater than previously predicted. Furthermore, evolutionary dynamics show considerable nonlinearities before settling at an ESS. The nonlinear effects depend on the starting conditions and indicate that evolutionary change is fastest when starting at an extremely biased sex ratio and that evolutionary change is weaker for parents of high condition. The results show neither a tendency to maximize average population fitness nor to minimize the deviation between offspring sex ratio and offspring reproductive value ratio.

The study highlights the importance of dynamic feedback in models of natural selection and provides a new methodological framework for analyzing the evolution of continuous strategies in structured populations.

## Graphical abstract

This study presents a model of the evolutionary dynamics of the Trivers–Willard effect in the Columbian ground squirrel (*Urocitellus columbianus*). Condition‐dependent offspring sex ratio is treated as a continuous function of maternal weight and modeled by means of a nonparametric spline function. Results show that population feedback produces complex evolutionary dynamics and changes the predicted Trivers–Willard effect.

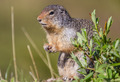

## 1 INTRODUCTION

The Trivers–Willard hypothesis (TWH) states that parents in good condition preferentially produce the sex with a higher variation in reproductive value, whereas parents in bad condition favor the opposite sex (Trivers & Willard, 1973). Many empirical studies support the TWH (Cameron & Dalerum, 2009; Cameron & Linklater, 2007; Charnov et al., 1981; Clutton‐Brock, Albon, & Guinness, 1984, 1986; Mealey & Mackey, 1990). However, there is a considerable amount of contradictory empirical evidence (Brown & Silk, 2002; Cameron, 2004; Hewison & Gaillard, 1999; Kolk & Schnettler, 2013; Sheldon & West, 2004). Schindler et al. (2015) show that these inconsistencies may arise because different life histories change the predictions of the TWH. To account for this, they generalize the TWH to any functional relationship between parental condition and offspring reproductive value: parents preferentially produce the sex that has the higher expected reproductive value, given the parents’ own condition. Thus, depending on the specific offspring reproductive value functions, different types of the Trivers–Willard effects (TWE) may emerge.

Since offspring reproductive value functions depend on the long‐term development of the whole population, this generalized version of the TWH requires an explicit model of the underlying population dynamics. Schindler et al. (2015) demonstrate how this can be accomplished by a two‐sex integral projection model (IPM; Ellner et al., 2016; Ellner & Rees, 2006). In contrast to previous studies, the demographic approach provides an explicit test of the conditions necessary for a TWE.

However, the predictions made by Schindler et al. (2015) only refer to the momentary change in condition‐dependent offspring sex ratio. This implies that offspring reproductive value is treated as an independent variable to predict the direction of change given an equal offspring sex ratio. It is not possible to infer the long‐term evolutionary dynamics from this momentary change. The main reason for this is that a biased sex ratio is known to influence male and female reproductive values (Fisher, 1930). Since every offspring has one mother and one father, the absolute reproductive value of males and females must be equal; given an unequal sex ratio, this implies that per capita reproductive value will be higher for the rarer sex (this is known as the Fisher condition). Consequently, if parents adjust their offspring sex ratio according to the generalized TWE, per capita male and female reproductive values will change, which, in turn, alters the expected TWE. This introduces a nonlinear feedback loop, which may change the empirical predictions derived from the model (Borgstede, 2019). Shyu and Caswell (2016) investigate the effect of such nonlinear feedback on the evolutionary dynamics of the TWE using a matrix population model. However, their model uses only two discrete stages to capture parental condition. Leimar (1996) acknowledges the relevance of changing reproductive values for the dynamics of the TWE for continuous parental condition. However, his formal analysis does not incorporate the effects of varying abundances of available mates on evolutionary dynamics.

This study aims at exploring the effects of nonlinear population feedback on the evolution of the TWE. I use a continuous two‐sex integral projection approach to model the long‐term evolutionary dynamics of the TWE. To accomplish this, I introduce a new method to model the evolution of continuous conditional strategies by means of nonparametric spline functions (Green & Silverman, 2000; Hastie & Tibshirani, 1999). I further modify the two‐sex IPM used by Schindler et al. (2015) such that dynamic evolutionary feedback can be incorporated by means of an evolutionary invasion analysis. Using a published model of the demography of the Columbian ground squirrel (*Urocitellus columbianus*), I simulate the evolutionary trajectories of condition‐dependent offspring sex ratio to find evolutionarily stable strategies (ESS) and investigate possible explanations for the resulting evolutionary endpoints.

## 2 MODEL

IPMs describe the dynamics of continuous trait distributions (like size or weight) in a population by projecting the population distribution from one time step to the next time step (Easterling et al., 2000):

(1) $$n\left(x,t+1\right)=\int \left[p(x,y)+q(x,y)\right]n\left(y,t\right)dy$$

The number (or relative frequency) of trait value x at time t+1 is denoted n(x,t+1). This trait frequency is described as a function of trait frequency y at time t, which is denoted n(y,t). The trait distribution in the next time step is calculated from the trait distribution in the previous time step by means of a survival function p(x,y), denoting the probability of an individual of trait value y to survive and develop into an individual of trait value x, and a fertility function q(x,y), denoting the contribution of a surviving individual of trait value y to the frequency of trait value x offspring.

Here, I use a slightly modified version of the two‐sex IPM introduced by Schindler et al. (2013), describing the relative trait distributions of males and females separately, such that $n_{m}(z,t)+n_{f}(y,t)=n(y,t)$. The survival functions are treated as in the one‐sex case, yielding $p(x,z)n_{m}(z,t)$ and $p(x,y)n_{f}(y,t)$, respectively. Fertility is described with regard to all possible trait combinations of female and male individuals as captured by the term $n_{f}\left(y,t\right)n_{m}\left(z,t\right)$. The probability that a trait value y female and a trait value z male mate is denoted m(y,z). This mating probability is multiplied by the number of offspring per breeding event for this combination of parental trait values, R(y,z). To obtain the contribution of a breeding event to the trait frequencies in the next time step, the result is multiplied by the conditional probability f(x|y,z) of an offspring having trait value x, given that the mother has trait value y and the father has trait value z. The two‐sex IPM can now be described by the dynamic equations for male and female trait values:

(2) $$n_{m}\left(x,t+1\right)=\int p\left(x,z\right)n_{m}\left(z,t\right)dz+C_{n_{f},n_{m}}\int s\left(y\right)f\left(x|y,z\right)m\left(y,z\right)n_{f}\left(y,t\right)n_{m}\left(z,t\right)R\left(y,z\right)dy\;dz$$

(3) $$n_{f}\left(x,t+1\right)=\int p\left(x,y\right)n_{f}\left(y,t\right)dy+C_{n_{f},n_{m}}\int \left(1‐s\left(y\right)\right)f\left(x|y,z\right)m\left(y,z\right)n_{f}\left(y,t\right)n_{m}\left(z,t\right)R\left(y,z\right)dy\;dz$$

Here, s(y) denotes the proportion of male offspring as a function of maternal weight y and 1‐s(y) the corresponding proportion of female offspring. $C_{n_{f},n_{m}}$ is a normalization constant that ensures that the overall number of birth events is set equal to the total number of reproducing females, that is, every female above a threshold rate has an expectation of reproduction:

(4) $$C_{n_{f},n_{m}}=\frac{\int_{y_{\min}}^{\infty }n_{f}\left(y,t\right)dy}{\int_{0}^{\infty }m\left(y,z\right)n_{f}\left(y,t\right)n_{m}\left(z,t\right)dy\;dz}$$

with $y_{\min}$ being the minimum trait value of reproducing individuals.

To model the effect of male condition on reproductive value, the mating function was chosen such that males with high trait values have a higher probability of mating than males with lower trait values. Following Schindler et al. (2013), the mating function was

(5) $$m(y,z)=\left\{\begin{array}{l} 1/2e^{\rho z},\text{if}\;y,z\geq y_{\text{min}}\\ 0\;\text{otherwise} \end{array}\right.$$

with ρ specifying the degree of male mating advantage and $y_{\min}$ being the minimum trait value of reproducing individuals.

In this study, I use a published two‐sex IPM of the Columbian ground squirrel (*Urocitellus columbianus*) with body weight as the conditioning variable. Columbian ground squirrels exhibit a polygynandrous mating system where larger males show strong territorial behavior. Although territorial behavior does not guarantee mating success, it raises the chances of a male to obtain the first copulation from estrous females residing in its territory (Manno & Dobson, 2008). However, some males adopt a nonterritorial mating strategy, thereby obtaining mating opportunities without having to defend a territory (Balmer, 2010). Therefore, it is difficult to predict whether it is profitable for females to skew their offspring sex ratio conditioned on weight. A long‐term study by Gedir and Michener (2014) revealed no condition‐dependent offspring sex ratio in a related species (*Urocitellus richardsonii*). Hence, it is not clear what to expect with regard to the TWE for the Columbian ground squirrel.

The functions p(x,y), R(y,z), f(x|y,z), and s(y) were approximated by generalized regression models based on data collected between 1994 and 1998 in the Sheep River Provincial Park in the foothills of the Rocky Mountains in Alberta, Canada. Data included female weight after spring emergence, female survival to the next year, the weight of female survivors the next spring, litter size at weaning and offspring weights at weaning. Since male and female squirrels have similar demographic characteristics, the estimated functions were used to describe female and male demography. Further details on the model and its parameterization can be found in Schindler et al. (2013). The model was chosen to enable a direct comparison between the dynamic approach taken in this study and the predictions made by Schindler et al. (2015). The model was implemented using R, version 4.0.3 (R Core Team, 2020). All functions and intermediate results were double‐checked with the results of Schindler et al. (2013) and validated using the original MATLAB script used in Schindler et al. (2015).

## 3 METHOD

The demographic model was used to perform an evolutionary invasion analysis. The rationale behind this approach is that evolutionary change can be analyzed by modeling successional invasions in a monomorphic population. As long as we consider only small changes, a rare mutant phenotype that outperforms the resident phenotype in terms of long‐term growth rate under the conditions established by the resident population will eventually replace the resident phenotype (Dercole & Rinaldi, 2008).

The first step of the analysis consists of calculating the long‐term population growth rate λ and the stable stage distribution of the resident model. This is accomplished by iterating the demographic model until the relative weight distribution does no longer change. A numerical approximation of the integral was used to project the population to the next generation. The second step was to calculate the long‐term population growth rate of the mutant model, given the stable stage distribution of the resident population. For this sake, a projection matrix was constructed, treating the contributions of male and female parents to the male and female population at time t+1 separately and then stacking the submatrices together (see Appendix S1 for technical details). This projection matrix was substituted with the abundances of reproducing males and females under the conditions established by the resident phenotype, thereby fixing the mating rates. The mutant growth rate was obtained by calculating the dominant eigenvalue of the mutant projection matrix (Caswell, 2001). The difference between mutant growth rate $\lambda^{\prime }$ and resident growth rate λ determined the invasion fitness $w(s^{\prime },s)$ of the mutant strategy $s^{\prime }$ with respect to the resident strategy s.

Condition‐dependent reproductive values were obtained by substituting the model equations of the resident model with the stable stage distribution and calculating the left eigenvector of the dominant eigenvalue of the asymptotic projection matrix. Offspring reproductive values for mothers and fathers were obtained by multiplying the weight‐dependent reproductive values for males and females by the expected male and female offspring weight distributions. Male and female offspring reproductive values ($v_{m}$ and $v_{f}$) were compared by dividing male reproductive value by the sum of male and female reproductive value to form a reproductive value ratio (RVR). Conditioning offspring reproductive values of maternal weight, we can define a corresponding reproductive value ratio function RVR(y):

(6) $$\text{RVR}\left(y\right)=\frac{v_{m}\left(y\right)}{v_{m}\left(y\right)+v_{f}\left(y\right)}$$

Hence, a RVR of 0.5 means that for a given weight, male and female offspring have the same reproductive value. Reproductive value ratios are preferred over differences (as in Schindler et al., 2015) because ratios are independent of arbitrary factors introduced by the choice of measurement units. By using RVR rather than the difference between male and female reproductive values, reproductive values are scaled such that they form a dimensionless number between zero and one. This allows for a direct comparison between reproductive values and sex ratios.

There are two standard approaches to model the evolution of continuous phenotypes such as condition‐dependent offspring sex ratio. First, one may specify the model for a small number of discrete levels of the conditioning variable (e.g., “high quality” and “low quality”). Second, one may describe the evolving strategy by means of a parametric function and then model natural selection on the parameters of this function (e.g., the slope and intercept of a linear function). However, both approaches limit the scope of analysis by introducing arbitrary restrictions on the phenotypic range of the evolving strategy.

To avoid the limitations that come with the above types of analysis, I model the evolving offspring sex ratio as a *nonparametric function* of adult condition. This allows for an unconstrained variation of the shape of the conditional offspring sex ratio function. Technically, this is accomplished by introducing small disturbances into the conditional offspring sex ratio using a Gaussian error distribution. The resulting disturbed strategy is then smoothed by a nonlinear spline function (Green & Silverman, 2000; Hastie & Tibshirani, 1999).

Because the two‐sex model used in the simulation is frequency‐dependent, it may well be nonergodic. This means that different initial conditions do not necessarily result in the same evolutionary endpoint. To assess nonergodicity, several simulations were performed with different initial population structures. The first variant starts at an equal offspring sex ratio for all mothers (the *equal SR* strategy). The second and third variants start at the maximum possible TWE with mothers producing exclusively one sex when below a certain weight and the other sex when above a certain weight, with a smooth but steep transition between male bias and female bias around a certain threshold. Since there is no a priori reason to expect a TWE in one direction or the other for Columbian ground squirrels, two starting positions for the maximum TWE were realized: one with low‐weight females producing exclusively females and high‐weight females producing exclusively males (classical, or *positive TW* strategy), and one with low‐weight females producing exclusively males and high‐weight females producing exclusively females (reversed, or *negative TW* strategy). Finally, the simulation was repeated with an initial population that almost exclusively produced males (the *male bias* strategy) and with an initial population that almost exclusively produced females (the *female bias* strategy).

For each of the starting conditions, evolutionary dynamics were modeled as a series of successive invasions, until the evolving offspring sex ratio strategy reached a stable equilibrium state (i.e., until small deviances from the established strategy consistently resulted in selection back toward the established strategy). Mutation rates and smoothing parameters for the spline function were calibrated such that small disturbances would alter the shape of the function without producing abrupt changes.^1^ Reproductive values and stable stage distributions were calculated for all invading strategies using a numerically approximated population projection matrix (see Appendix S1). For each simulated invasion, the deviance D between offspring reproductive value ratio, which is given by RVR(y), and offspring sex ratio, which is given by s(y), was calculated as the integral over the squared difference between the two values ranging over maternal weight:

(7) $$D=\int_{y_{\min}}^{\infty }{\left[s(y)‐\text{RVR}(y)\right]}^{2}dy$$

with $y_{\min}$ being the minimum trait value of reproducing individuals.

## 4 RESULTS

**FIGURE 1:**
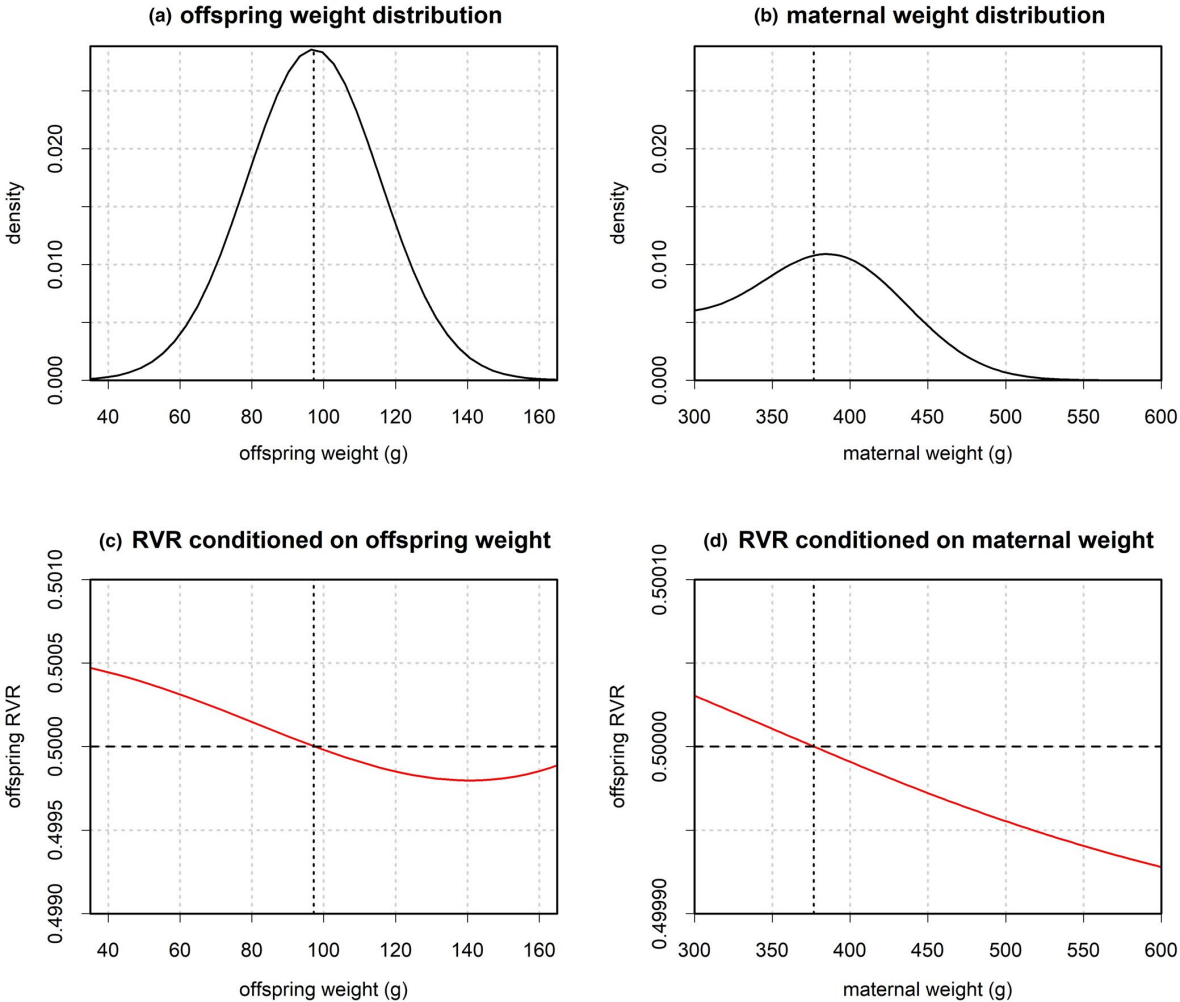
Weight distributions and reproductive value ratios (RVR) of the equal sex ratio model. Offspring weight (panel a) is a function of parental weight with an overall expected value of 97.2 g. Panel b presents the stable weight distribution for reproducing females at equilibrium. The expected value of maternal size is 376.94 g. Individuals below this value produce, on average, offspring that weigh less than the expected value of the offspring distribution; individuals above this value produce, on average, offspring that weigh more than the expected value of the offspring distribution. Panels c and d depict the offspring RVR as a function of offspring weight (panel c) and maternal weight (panel d). Panel d corresponds to a reversed TWE with RVR biased toward males below average maternal weight and RVR biased toward females above average maternal weight. This is a direct consequence of the pattern depicted in panel c, where the RVR is biased toward males below average offspring weight and the RVR is biased toward females above average offspring weight

Figure 1 depicts the main results obtained from the reference model (equal SR). The long‐term growth rate λ of the reference model was 1.05 and thus matched the one reported in Schindler et al. (2013). The stable stage distribution revealed that, at equilibrium, mean maternal weight was 376.94 g (Figure 1b). Expected mean offspring weight at equilibrium was 97.2 g (Figure 1a). Offspring RVR was calculated as a function of offspring weight (Figure 1c) and as a function of maternal weight (Figure 1d). Conditioned on offspring weight, the RVR had a reversed s‐shape with an equal sex ratio at the point of mean offspring weight. Below this point, males had a higher reproductive value than females. Above this point, females had a higher reproductive value than males. Conditioned on maternal weight, the RVR was a monotone decreasing function of weight with an equal sex ratio occurring at the point of mean maternal weight. The reason why an equal sex ratio occurs at the point of mean maternal weight is that the inheritance function f(x|y,z) yields average‐sized offspring for average‐sized mothers. Hence, the expected offspring RVR given maternal weight depicted in Figure 1d mirrors the overall shape of the offspring RVR depicted in Figure 1c. This matches the results in Schindler et al. (2015) (note that here I use reproductive value ratios rather than differences). The negative slope of the RVR results from the effects of the male mating advantage on the demography of the population. Given the parameterization from Schindler et al. (2013), mating success for females is constant once they have reached the minimum weight for reproduction. However, males that have just reached the minimum weight for reproduction have a considerably lower chance to mate when compared to females, whereas high‐weight males have a higher chance to reproduce when compared to females. Consequently, because males need to reach a certain weight to reproduce effectively, high‐weight mothers benefit more from producing females who start reproduction at a younger age (Schindler et al., 2015).

**FIGURE 2:**
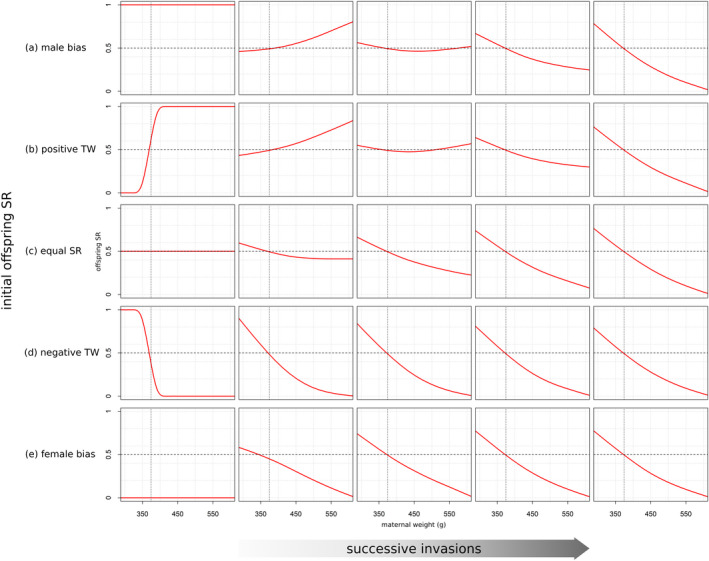
Evolving offspring sex ratio s(y) (proportion of males among offspring) as a function of maternal condition y (weight in g). Each row corresponds to a different initial conditional offspring sex ratio, which is depicted on the first panel from the left in each row. The rows depict the conditional offspring sex ratios that evolve over the course of several thousand successional invasions. The dotted vertical lines mark the average weight of reproducing females. Despite different initial values and different intermediate steps, all simulations eventually settle at the same evolutionary equilibrium, which can be seen in the last column on the right‐hand side

Figure 2 illustrates the evolutionary change in weight‐dependent offspring sex ratio as a series of successional invasions for each of the specified starting conditions. Within the range of error introduced by the numerical approximations, all simulations of evolutionary dynamics converged toward a single evolutionarily stable strategy. As predicted from the reference model, at the ESS, condition‐dependent offspring sex ratio was male‐biased below average maternal weight and female‐biased above average maternal weight.^2^ However, contrary to the results of Schindler et al. (2015), who predicted a *small* reversed TWE, the effect is indeed very large, with a strong male bias for low‐weight mothers and exclusive production of females for extremely high‐weight mothers.

Figure 2 shows that the evolved evolutionary equilibrium is independent of the initial state of the population. However, the simulations do not always produce a “smooth” transition from the starting point toward the ESS, but predict considerable nonlinear dynamics, depending on the initial offspring sex ratio. Most strikingly, the rate of change in condition‐dependent offspring sex ratio appears to be higher for small‐weight and medium‐weight mothers than for high‐weight mothers. This effect is most obvious in the male bias condition (Figure 2a), where low‐weight and medium‐weight mothers rapidly adjust their offspring sex ratio away from the (evolutionary unfavorable) extreme excess in males, whereas high‐weight mothers seem to be less affected and retain a strong male bias at first. Thus, in the male bias condition, selection first leads to a positive TWE (i.e., female bias for low‐weight mothers and male bias for high‐weight mothers), which is later reversed until it gradually converges toward the ESS. The same overall pattern is also visible in the positive TWE condition (Figure 2b), where the offspring sex ratio of low‐weight mothers changes rapidly away from the initial extreme female bias, while high‐weight mothers are less affected by selection at first. Even in the equal sex ratio condition (Figure 2c), selection toward the ESS is slightly lagged for high‐weight mothers, before it starts to change rapidly toward a female‐biased offspring sex ratio.

**FIGURE 3:**
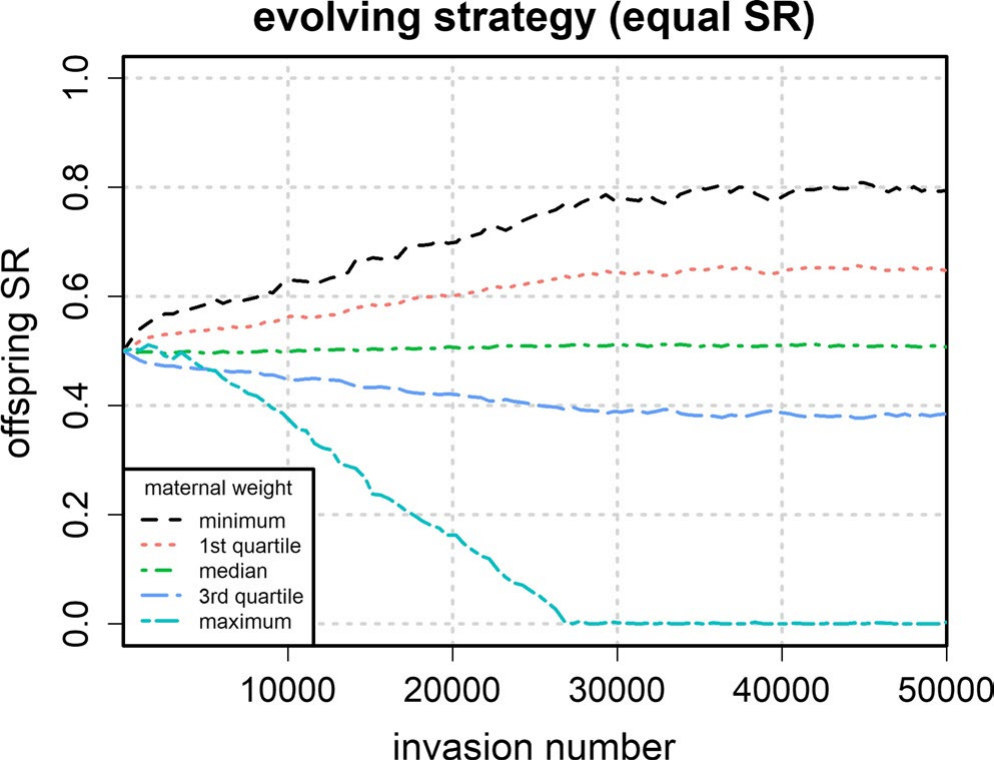
Evolutionary trajectories of offspring sex ratio s(y) (proportion of males among offspring) as a function of maternal condition y (weight in g) for the equal sex ratio condition. Evolutionary dynamics were modeled as successive mutant invasions starting at an equal offspring sex ratio for all maternal weights. The different lines depict the evolutionary trajectories of the quartile values (calculated from the maternal weight distribution of the reference model)

**FIGURE 4:**
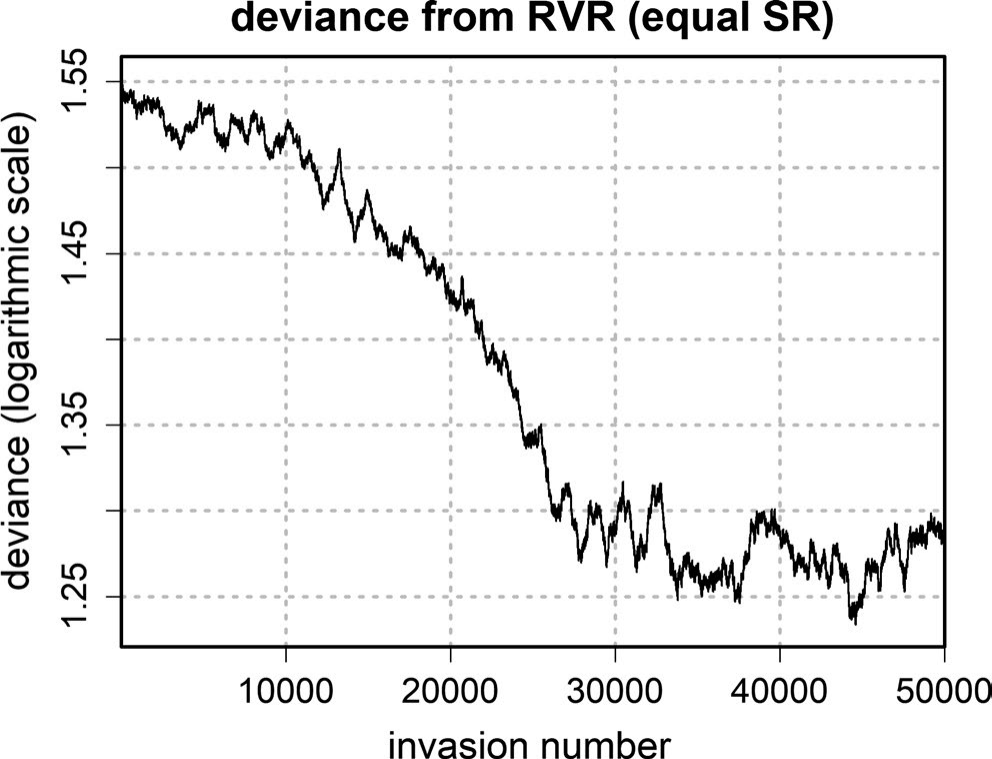
Deviance between offspring SR and offspring RVR (moving average over 1,000 iterations) over the whole range of successional invasions for the equal sex ratio condition. Deviance was scaled using a logarithmic transformation

**FIGURE 5:**
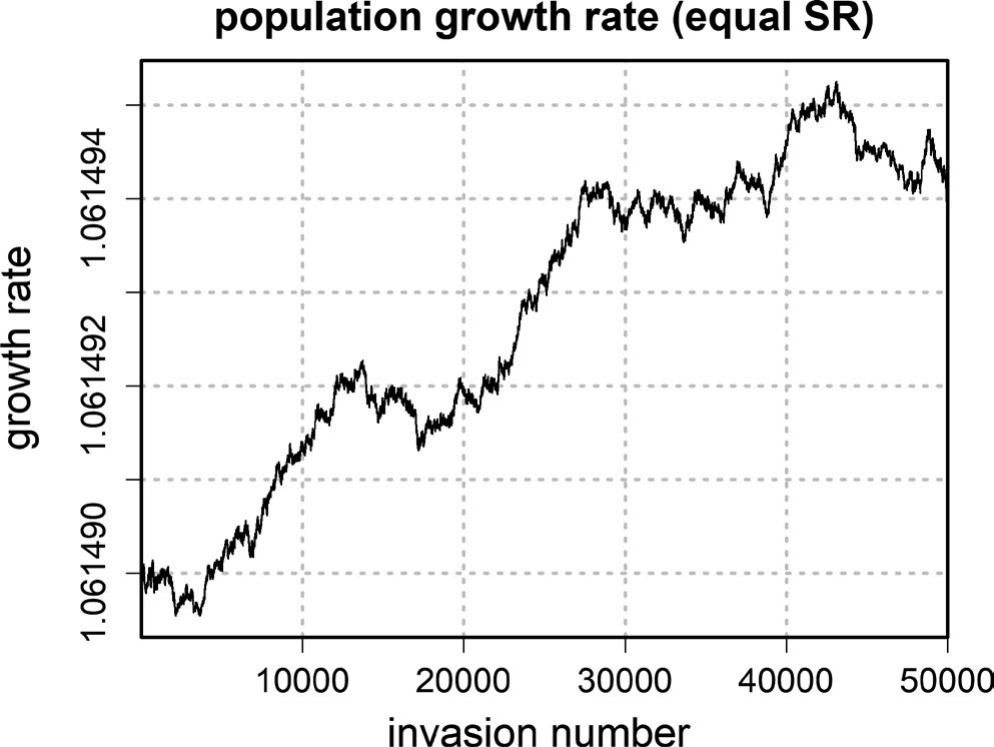
Population growth rate λ (moving average over 1,000 iterations) over the whole range of successional invasions for the equal sex ratio condition

**FIGURE 6:**
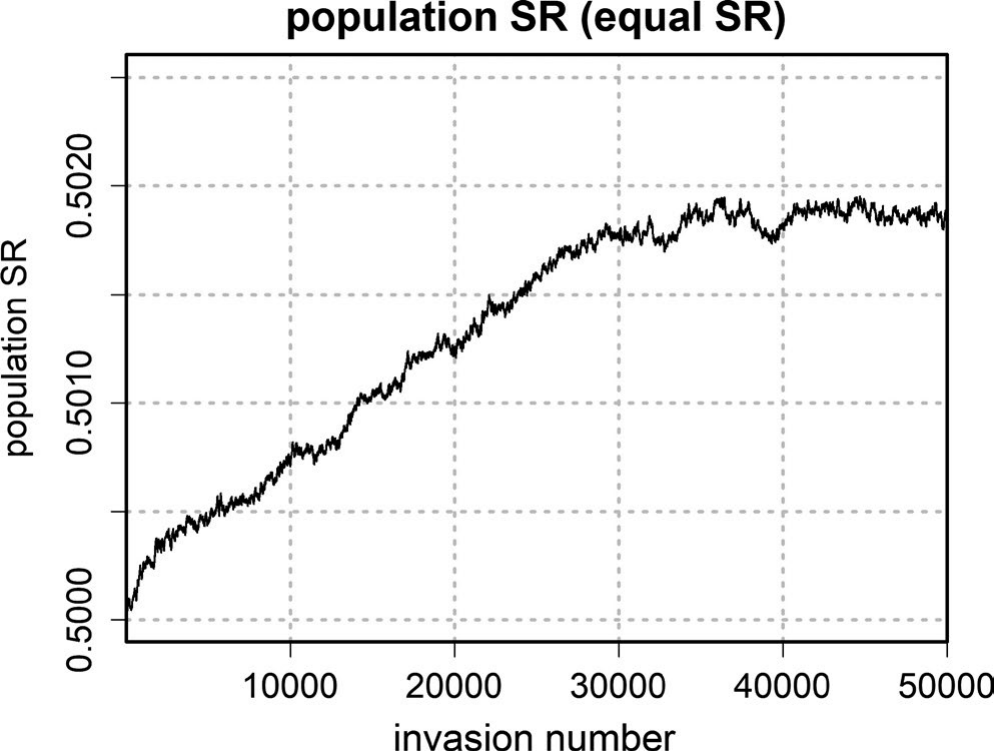
Population sex ratio (moving average over 1,000 iterations) over the whole range of successional invasions for the equal sex ratio condition. Sex ratio was measured as the proportion of males in the overall population

To investigate the full evolutionary dynamics that lead to the ESS, the evolutionary trajectories of condition‐dependent offspring sex ratio were analyzed using the quartiles of maternal weight from the reference model (i.e., for minimum, maximum, and median, as well as the 25th and 75th percentiles of the maternal weight distribution). Additionally, the trajectories of the corresponding deviance between offspring RVR and offspring SR, the population growth rate, and the population sex ratio were tracked for all conditions. Figures 3, 4, 5, 6 depict the corresponding dynamics over the whole range of the successional invasions for the equal SR condition. The corresponding plots for the remaining four conditions can be found in Appendix S2.

Figure 3 depicts the evolutionary dynamics of condition‐dependent offspring sex ratio for all quartile values of maternal weight starting at an equal offspring sex ratio (equal SR strategy). Like in Figure 2, the rate of change appears to be lagged for the maximum weight class, especially when compared to the 3rd quartile weight class. Whereas during the first 100 invasions, the rate of change is higher for the 3rd quartile weight class than for the maximum weight class, after a couple of hundred successive invasions, the highest weight class reaches the highest rate of change.

Following the logic of Schindler et al. (2015), expected offspring RVR should predict not only the direction but also the magnitude of the TWE. If this is correct, it appears plausible to assume that the evolving offspring sex ratio should eventually approach the offspring RVR, thereby reducing the deviance between both measures. In fact, the simulation reveals a tendency toward a smaller deviance, as can be seen in Figure 4, which shows a moving average of the scaled deviance^3^ over the range of invasions for the equal SR condition. As predicted, average deviance was higher at the beginning of the simulation when compared to the ESS. However, depending on the starting conditions, deviance did not always decrease monotonically with successive invasions.^4^

Figure 5 depicts the evolutionary trajectory of average fitness as measured by population growth rate λ for the equal SR condition. Although there seems to be an overall tendency toward higher population growth rate when starting from the equal SR strategy, the ESS did not coincide with the strategy that maximizes λ. Specifically, strategies with a strong bias toward female offspring produced higher population growth because, due to the scaling factor introduced in Equation (6), the overall number of matings equaled the number of reproducing females. Therefore, selection did not maximize average fitness.^5^

Figure 6 shows how the overall population sex ratio (male proportion of individuals) changes as a result of biased offspring sex ratios for the equal SR condition. The population sex ratio starts at 0.5 and gradually increases with successional invasions. At equilibrium, the population sex ratio is 0.502 and thus slightly skewed toward males. This is in line with Bull and Charnov (1988), who predict that, given condition‐dependent offspring sex ratio, there should be a small overall excess of the sex that is overproduced by mothers in bad condition.

## 5 DISCUSSION

The aim of this study was to explore the evolutionary dynamics of the TWE in a way that accounts for the mutual dependence between reproductive values and sex ratios and allows to model unconstrained selection on a continuous conditional phenotype. This was accomplished by means of an evolutionary invasion analysis in combination with a nonparametric approach to modeling offspring sex ratio as a continuous function of maternal condition.

The first main result was that the evolving TWE is much higher than predicted by the initial offspring reproductive values. This is caused by a positive feedback‐loop consisting of offspring sex ratio affecting offspring reproductive value, which then affects offspring sex ratio again. If, conditioned on weight, mothers produce more offspring of the sex with greater reproductive value, the relative abundances of males and females in the population change. This, in turn, changes the reproductive values. Additionally, by altering conditional offspring sex ratio, mothers directly affect the number of their own male and female offspring. Consequently, because offspring reproductive value is calculated over the sum of all offspring, this naturally biases the expected offspring reproductive values in the direction of the offspring sex ratio. Therefore, reproductive values obtained from a demographic model should be treated with care when they are used to predict biased sex ratios like in the case of the TWE. The results presented here show that neglecting this feedback loop may lead to erroneous conclusions.

The results further show that, although all simulations converged toward a single ESS, the evolutionary trajectory depends on the initial population structure. The general pattern implied by the simulations is that evolutionary change of condition‐dependent offspring sex ratio occurs faster for medium and low‐weight mothers and more slowly for extremely high‐weight mothers. This can be explained by the fact that there are generally only very few extremely heavy individuals in the population because the weight distribution tends to be skewed (compare Figure 1b). Consequently, although the marginal gain in individual fitness from a biased sex ratio is highest for mothers of extremely high weight, they hardly contribute to the overall population. Therefore, changes in offspring sex ratio for high‐weight mothers have a comparably small effect on population growth, and, thus, invasion fitness.

The lag in evolutionary change observed for high‐weight mothers can further drastically change the dynamics of the overall shape of the evolving strategy. This is most obvious in the male bias condition. Here, the initial population consists nearly exclusively of males. Due to the Fisher condition, mothers biasing their offspring sex ratio toward females will have a higher evolutionary fitness, which is why there is strong selection away from the male bias strategy. Because selection is weaker on high‐weight mothers, there will still be relatively many high‐weight males in the population, however. Consequently, low‐weight males will have a smaller mating rate than in the equal sex ratio condition because they cannot compete effectively with the high‐weight males. It thus becomes more profitable for low‐weight mothers to produce more females than males. This explains the initial positive TWE that can be observed in Figure 3a. However, due to the delayed reproduction for males (as compared to females of equal birth weight), producing females is also more profitable (even to a higher degree) for high‐weight mothers. Therefore, the initial TWE is eventually reversed as high‐weight mothers start to bias their offspring sex ratio away from males. These nonlinearities are a direct consequence of the dynamic population feedback introduced by changing reproductive values.

In addition to these results, it was found that selection tended to produce higher average fitness at the ESS when compared to the equal SR condition. However, the ESS did not coincide with the strategy that maximizes average fitness, because the mating function used in the simulations forces population growth to be proportional to the number of reproducing females. Consequently, average fitness would be highest, when the population sex ratio is skewed toward females. However, at the ESS, the overall population sex ratio was slightly skewed toward males as predicted by Bull and Charnov (1988). Hence, there was no general tendency to maximize average fitness (as measured by population growth rate). This may be a general pattern inherited from the nonlinearity of two‐sex models. Since the number of reproductive events is scaled according to the number of reproducing females at each iteration, the growth rate of rare mutants depends on the strategy that is followed by the resident population. Thus, the long‐term population growth rate of a specific strategy may differ from its growth rate when being a rare mutant in a monomorphic population. Therefore, in sexually reproducing species, evaluating different strategies by means of their long‐term population growth rates may give a misleading impression with regard to the direction of evolutionary change.

Furthermore, evolutionary dynamics reduced the deviance between offspring sex ratio and offspring reproductive value ratio. This means that at the ESS the condition‐dependent sex ratio strategy tended toward the ratio between the expected offspring reproductive values. This is in line with previous theoretical predictions stating that the TWE is to be understood as a condition‐dependent bias toward expected offspring reproductive values. For example, Schindler et al. (2015) base their predictions about the direction and magnitude of the TWE solely on expected offspring reproductive values, without testing whether the implied outcome of selection actually matches these predictions. The results of the current study show that evolutionary dynamics are not guaranteed to exhibit a general tendency to reduce the deviation between offspring sex ratio and offspring reproductive value ratio. One possible explanation for this effect could be that in the region between the maximal negative TWE and an equal offspring sex ratio, selection toward offspring RVR (as implied by the TWH) is opposed by selection toward an equal sex ratio (as implied by the Fisher condition), leading to an equilibrium that lies somewhere between the minimum deviance strategy and the equal sex ratio strategy. However, the results are not conclusive in this respect. Therefore, further research should investigate the effects of opposing selection pressures in the context of the TWH.

In addition to the results specific to the TWE, this study introduced some general methodological improvements over existing work. First, the definition of an explicit projection matrix to approximate the two‐sex IPM makes this type of model accessible for evolutionary invasion analysis. Second, the nonparametric approach to describe mutations in a continuous strategy makes it possible to model the dynamics of whole functions, rather than single parameters. This allows for the analysis of complex evolutionary processes without arbitrary (and often, biologically unjustified) assumptions about the functional form of the evolving phenotypes.

Some limitations of the study have to be mentioned. First, the successive invasion paradigm is only one possible way to model evolutionary dynamics. The underlying assumptions of a monomorphic population and rare mutations limit the scope of this study. Furthermore, although the simulations were performed using various starting conditions, this does not guarantee a general tendency toward the identified evolutionary endpoint. However, since the starting conditions cover all plausible extreme biases (as well as a no‐bias condition), it is difficult to conceive of an evolutionary trajectory that would not eventually converge with the observed ones. In addition, even when selection initially favored a positive TWE in the male bias condition, the trajectory eventually approached the predicted negative TWE. Taken together, this gives considerable evidence for a general tendency toward the observed ESS.

Another limitation stems from the choice of the smoothing method used in the simulations. Spline functions are only one way to generate a nonparametric function from a randomly disturbed set of data points. Thus, the evolving strategy was not completely independent of constraining conditions. Moreover, the amount of random noise cannot be chosen arbitrarily small because if disturbances are too small, they are smoothed by the spline function. As a result, the evolving sex ratio did not converge to an exact ESS but settled around an evolutionary endpoint with a (small but detectable) quasi random error distribution. This can be seen in Figure 3, where the lines show some unsystematic disturbances around the equilibrium point. Consequently, the predicted ESS is merely an approximation within the scope of the constraints imposed by the choice of the smoothing method. However, these constraints are certainly much less influential than those of the parametric approach usually applied to model conditional strategies. Despite these limitations, the results yield important insights into the evolutionary dynamics of the TWE on a theoretical level and help to understand how condition‐dependent sex ratio evolves in species with a complex life history.

This study shows that understanding the conditions of the TWE is even more difficult than previously thought and that the evolutionary dynamics of the TWE may be highly nonlinear depending on the starting conditions. While it is necessary to estimate condition‐dependent offspring reproductive values from demographic data, these are not sufficient to predict the direction and strength of the TWE. The results presented here show that considering evolutionary dynamics and population feedback is essential to understand how condition‐dependent sex ratios evolve as the result of a complex interplay between natural selection and demography.

## CONFLICT OF INTERESTS

The author declares that the research was conducted in the absence of any commercial or financial relationships that could be construed as a potential conflict of interest.

## AUTHOR CONTRIBUTIONS

**Matthias Borgstede:** Conceptualization (lead); Formal analysis (lead); Investigation (lead); Methodology (lead); Validation (lead); Visualization (lead); Writing‐original draft (lead); Writing‐review & editing (lead).

## Supporting information

Appendices S1 and S2

## Data Availability

The simulation code used in this study can be accessed via the Open Science Framework: https://doi.org/10.17605/OSF.IO/93KD8.

## ENDNOTES

^1^
In all simulations, the spline function was constructed using 15 equidistant knots and a Gaussian error distribution with μ=0 and σ=0.0005 (i.e., an average deviation of only five thousandths of a gram was allowed). The penalty for abrupt changes was calculated using *R*’s smooth.spline function with a smoothness parameter of $s_{\text{par}}=0.3$. Changing these parameters slightly altered the slope of the equilibrium strategy but did not affect the overall pattern of evolutionary dynamics observed in the simulations.

^2^
To double check the plausibility of this result, the simulation was repeated under random mating. Without male mating advantage, an equal offspring SR turned out to be evolutionarily stable (i.e., small deviations could not replace the equal SR strategy).

^3^
The deviance was scaled such that the range of values could be more easily compared across conditions. First, the natural logarithm of the deviance was calculated. Then, the minimum value of the logarithmized deviance across all conditions was subtracted. Finally, the resulting value was again logarithmized.

^4^
The strategy with the smallest deviance occurred within the first 800 invasions starting from the negative TW condition, which was later replaced by invading strategies with a greater deviance (compare Figure A2 in Appendix S2).

^5^
Compare Figure A3 in Appendix S2.
