# Supplementary material for: Evolutionary dynamics of the Trivers–Willard effect: A nonparametric approach
Source: Ecol Evol. 2021 Aug 17;11(18):12676–85. doi: 10.1002/ece3.8012 (PMC8462157; doi:10.1002/ece3.8012)
Supplement: Supplementary file 1 — Appendices S1 and S2 [file ECE3-11-12676-s001.docx]

# Appendix S1: Projection matrix of a two-sex IPM

A projection matrix $A$ was defined by separating the contributions from male adults at time $t$ to the male and female trait distributions at time $t+1$ from the corresponding contributions of female adults. The projection matrix is the sum of the transition matrix $T$ and the fertility matrix $K$:

|  | $A=T+K$ | ( 1 ) |
| --- | --- | --- |

Let $T_{m,m}$ be the contribution of surviving males to the male population, and $T_{f,f}$ the contribution of surviving females to the female population at $t+1$. The transition matrix then becomes:

|  | $T=\left( \begin{matrix} T_{m,m} & 0 \\ 0 & T_{f,f} \end{matrix} \right)$ | ( 2 ) |
| --- | --- | --- |

Because individuals do not change their sex, the contributions from surviving males to the female population and from surviving females to the male population are specified by null-matrices. The entries in the sub-matrices $T_{m,m}$ and $T_{f,f}$ are calculated using the survival and growth functions of the two-sex IPM given by $p\left( x,z \right)$ and $p\left( x,y \right)$, respectively.

The entries of the fertility matrix $K$ are calculated using the corresponding fertility components of the two-sex IPM. The contributions of male and female parents to the number of offspring at each trait value $x$ are separated by assigning half of the produced offspring to each of the two parents. Let $K_{m,m}$ be the contribution of reproducing males to the male population, $K_{f,m}$ the contribution of reproducing females to the male population, $K_{m,f}$ the contribution of reproducing males to the female population and $K_{f,f}$ the contribution of reproducing females to the female population at $t+1$. The corresponding fertility matrix is:

|  | $K=\left( \begin{matrix} K_{m,m} & K_{f,m} \\ K_{m,f} & K_{f,f} \end{matrix} \right)$ | ( 3 ) |
| --- | --- | --- |

The entries of the sub-matrices are calculated by means of the fertility components of the two-sex IPM given by:

|  | $K_{m,m}=C_{n_{f},n_{m}}\int\frac{1}{2}s\left( y \right)f\left( x \vert y,z \right)m\left( y,z \right)R\left( y,z \right)n_{f}dy$ | ( 4 ) |
| --- | --- | --- |
|  | $K_{f,m}=C_{n_{f},n_{m}}\int\frac{1}{2}s\left( y \right)f\left( x \vert y,z \right)m\left( y,z \right)R\left( y,z \right)n_{m}dz$ | ( 5 ) |
|  | $K_{m,f}=C_{n_{f},n_{m}}\int\frac{1}{2}(1-s\left( y \right))f\left( x \vert y,z \right)m\left( y,z \right)R\left( y,z \right)n_{f}dy$ | ( 6 ) |
|  | $K_{f,f}=C_{n_{f},n_{m}}\int\frac{1}{2}(1-s\left( y \right))f\left( x \vert y,z \right)m\left( y,z \right)R\left( y,z \right)n_{m}dz$ | ( 7 ) |

The two-sex IPM can now be approximated to an arbitrarily close degree using the following matrix population model:

|  | $n(t+1)=A\left( t \right)n(t)$ | ( 8 ) |
| --- | --- | --- |

The entries of the population vector $n$ are provided by $\int n_{m}dz$ for the male stages and $\int n_{f}dy$ for the female stages. The dominant eigenvalue of the matrix $A$ corresponds to the long-term population growth rate $\lambda$. The corresponding right eigenvector corresponds to the stable stage distribution, and the corresponding left eigenvector corresponds to the stage reproductive values.

# Appendix S2: Supplementary figures


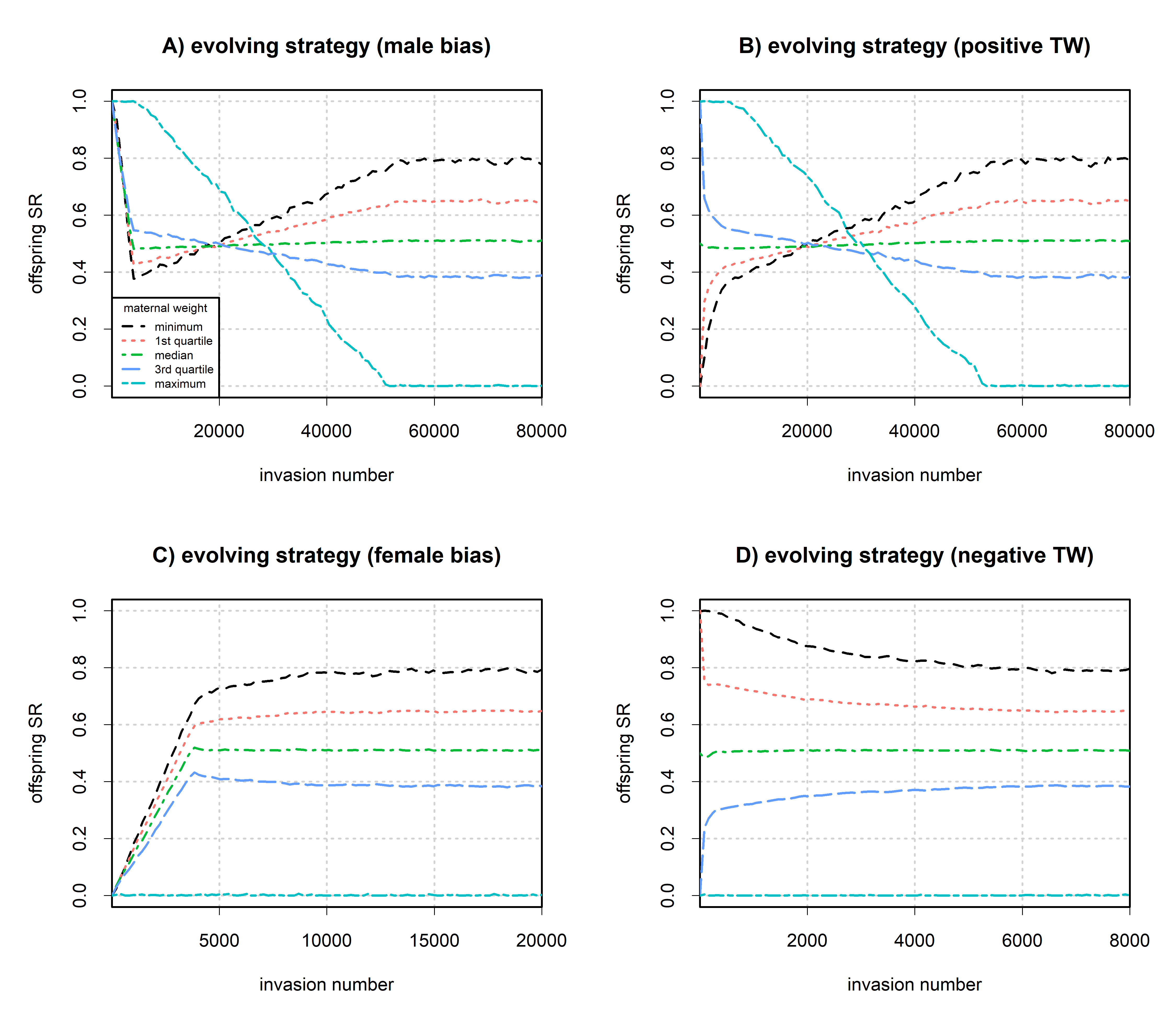


*Figure A1: Evolutionary trajectories of offspring sex ratio* $\boldsymbol{s(y)}$ *(proportion of males among offspring) as a function of maternal condition* $\boldsymbol{y}$ *(weight in g) for different starting conditions (male bias, female bias, positive TW, negative TW). Evolutionary dynamics were modelled as successive mutant invasions starting at an equal offspring sex ratio for all maternal weights. The different lines depict the evolutionary trajectories of the quartile values (calculated from the maternal weight distribution of the reference model).*


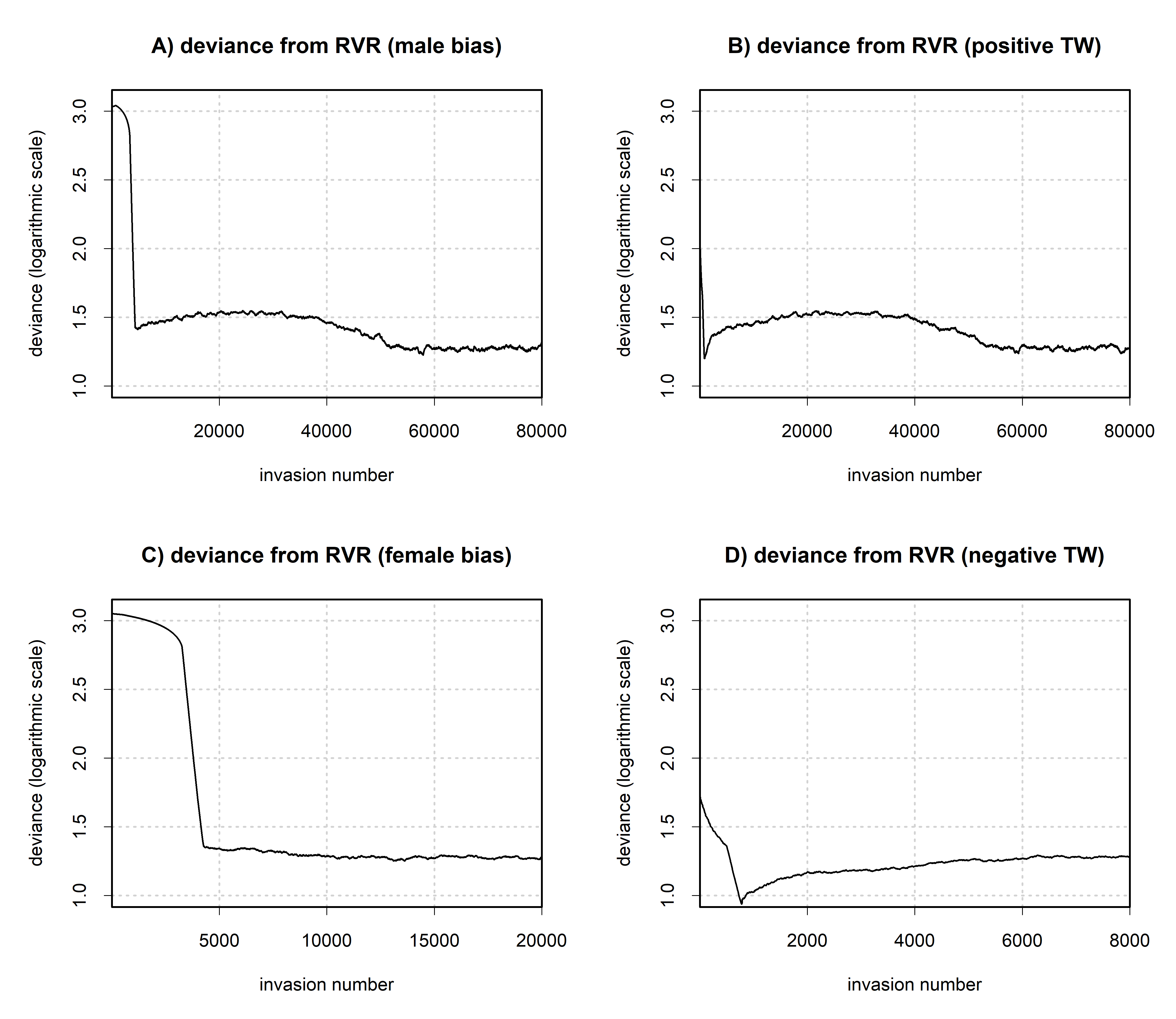


*Figure A2: Deviance between offspring sex ratio and offspring RVR (moving average over 1000 iterations) over the whole range of successional invasions for different starting conditions (male bias, female bias, positive TW, negative TW). Deviance was scaled using a logarithmic transformation.*


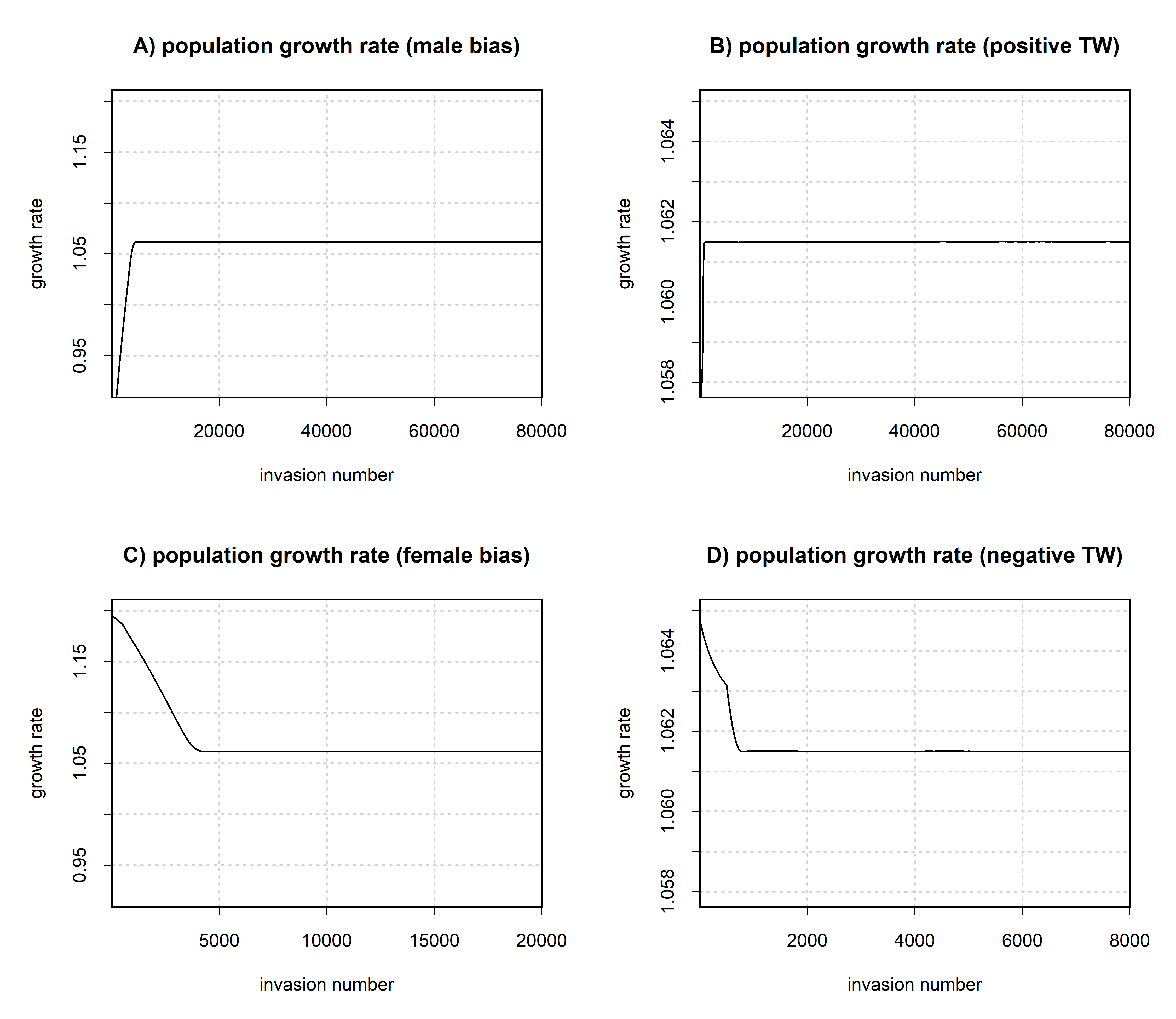


*Figure A3: Population growth rate* $\boldsymbol{\lambda}$ *(moving average over 1000 iterations) over the whole range of successional invasions for the equal sex ratio condition.*


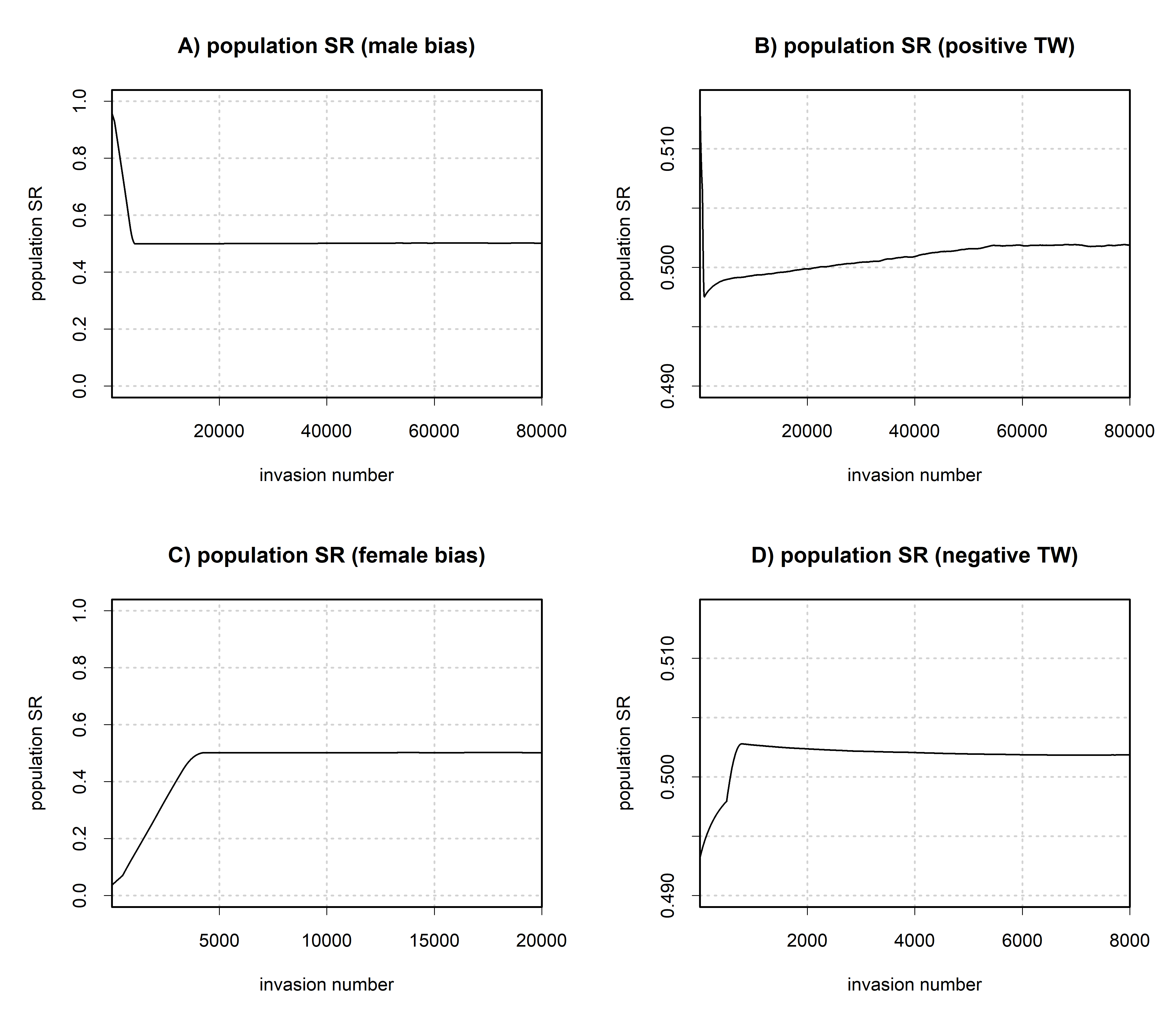


*Figure A4: Population sex ratio (moving average over 1000 iterations) over the whole range of successional invasions for different starting conditions (male bias, female bias, positive TW, negative TW). Sex ratio was measured as the proportion of males in the overall population.*
